# Editorial: Robotics in Extreme Environments

**DOI:** 10.3389/frobt.2021.744092

**Published:** 2021-08-19

**Authors:** Chie Takahashi, Manuel Giuliani, Barry Lennox, William R. Hamel, Rustam Stolkin, Claudio Semini

**Affiliations:** ^1^ Department of Psychology, University of Cambridge, Cambridge, United Kingdom; ^2^ University of the West of England, Bristol, United Kingdom; ^3^ The University of Manchester, Manchester, United Kingdom; ^4^ The University of Tennessee, Knoxville, TN, United States; ^5^ University of Birmingham, Birmingham, United Kingdom; ^6^ Dynamic Legged Systems, Istituto Italiano di Tecnologia (IIT), Genova, Italy

**Keywords:** robotics, manipulation, autonomous systems, remote operation, human-robot interaction, sensing

## Introduction

**FIGURE 1:**
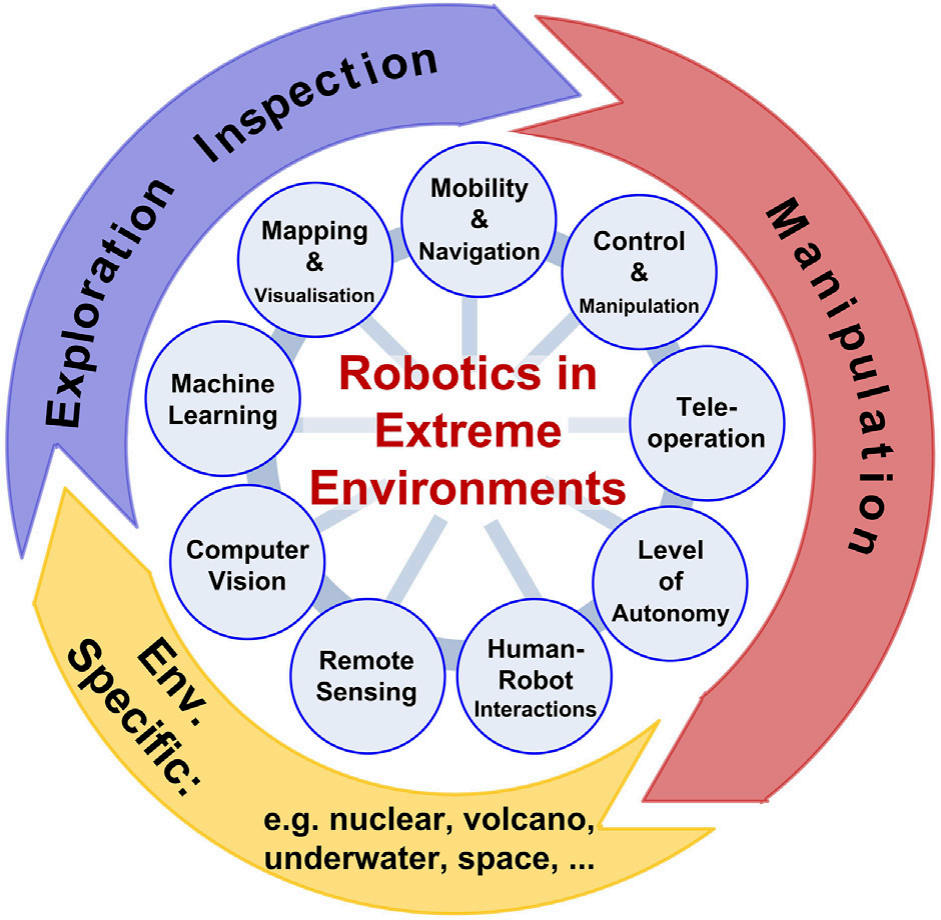
Overview of research topics and challenges for Robotics in Extreme Environments.

The development and deployment of robotics technology in extreme environments, such as nuclear decommissioning, offshore maintenance, space exploration and deep mining has received considerable attention in recent years. In all of these areas, robots are required to reduce the risks associated with operations staff, typically by removing the requirement for people to enter the hazardous environments and to increase productivity in high consequence and cluttered facilities. There has been significant effort in robotics research in this area, for example to make robotic systems able to survive in the presence of high levels of radiation and toxic substances, to operate at extreme pressures and temperatures, and to complete tasks safely in unstructured environments. We have seen there are many cross-domain challenges that researchers in robotics in extreme environments are working on (Figure 1).

In the context of robotics in extreme environments we raised the research topic aiming to bring together the latest cutting-edge research in the field, to deepen the current understanding and to share research challenges. This e-book comprises a collection of eight articles, published by Frontiers in Robotics and AI on the topic.

## Overview of the Contents of the E-Book

### Let’s Push Things Forward: A Survey on Robot Pushing

Stüber et al. present an overview of the current state of the art in robot pushing. Pushing objects is a crucial skill that mobile robots require for a wide range of applications, including tasks in extreme environments. The authors compared more than 50 publications dividing them into six categories: purely analytical, hybrid, dynamic analysis, physics engine, data driven and deep learning. A special focus was given to the problem of motion prediction of the object to be pushed. The authors conclude the paper with final remarks including open problems.

### Radiation Tolerance Testing Methodology of Robotic Manipulator Prior to Nuclear Waste Handling

Zhang, et al., present a radiation tolerance testing methodology for robotic manipulators, covering key aspects of the process from emulation of the radiation environment through to hot cell testing set up, data acquisition and analysis. The group from the Universities of Bristol and Manchester applied their methodology to a KUKA robotic arm and showed that it was surprisingly robust to radiation. These results suggest that the full spectrum of modern industrial robot products may be viable (with appropriate modifications) for practical use in future nuclear remote operations.

### A Holistic Approach to Human-Supervised Humanoid Robot Operations in Extreme Environments

The paper (Wonsick et al.) written by researchers from Northeastern University and Irish Manufacturing Research gives attention to the physical interaction aspects of a humanoid using glove box ports in terms of the mechanics of surfaces, footing and extended-arm operations. The authors present their initial concepts and ideas about making humanoid operations human-supervised along the lines that will make sense for glove box operations and provide pragmatic insights into how humanoid robots will need to be extended to 1 day be used in extreme environments.

### Radiation Mapping and Laser Profiling Using a Robotic Manipulator

Characterisation of nuclear materials, particularly in legacy facilities, is of great importance and in this article (White et al.), researchers from the University of Bristol, working with KUKA Systems, developed a robotic system, integrated with a radiation detector and time of flight sensor to provide measures of the distance to any object being scanned and the level of gamma activity at this location. By using the manipulator to raster over the surface of an object, the proposed technique is able to produce 3-dimensional radiation characterisation maps.

### Radiological Mapping of Post-Disaster Nuclear Environments Using Fixed-Wing Unmanned Aerial Systems: A Study From Chernobyl

This article (Connor et al.) describes a study led by researchers from the University of Bristol into the use of a fixed-wing unmanned aerial system to map radioactive contamination across relatively large areas of land. The capabilities of the proposed system were demonstrated through deployment within the Chernobyl Exclusion Zone (CEZ), where it generated radiation dose-rate maps of large areas of land that were consistent with more expensive, manual surveys.

### BVLOS Unoccupied Aerial Systems Operations in Highly-Turbulent Volcanic Plumes

Wood et al. provide insights into operating Unoccupied Aerial Systems (UAS) in highly-turbulent volcanic plumes. They present a detailed analysis of three missions in which the team flew a fixed wing UAS beyond visual line of sight into the plumes of Manam volcano, Papua New Guinea. The paper contains a detailed description of the used UAS and provides insights into plume sampling applications, with the authors giving recommendations for physical parameters and propulsion systems of aircraft used for taking measurements in turbulent volcanic plumes.

### Automatic Fracture Characterization Using Tactile and Proximity Optical Sensing

Palermo et al. present a custom-designed integrated tactile and proximity sensor that can be used for automatic detection of surface cracks. This approach might be more suitable for operation in extreme environments where, for example, radiation may damage electronic components of commonly employed sensing devices. For the detection, the sensor slides across different surfaces and records data. Using machine learning, the team can then classify fractures and other mechanical features with an average crack detection accuracy of ∼94% and width classification accuracy of ∼80%.

### Simultaneous Material Segmentation and 3D Reconstruction in Industrial Scenarios

Zhao et al. present a novel transfer learning approach for material segmentation and categorization of RGB images, with a special focus on nuclear waste. The authors combine transfer learning with Recurrent Neural Networks to perform boundary-aware annotation and 3D semantic reconstruction. Additionally, the authors generated a new dataset that includes RGB image patches and fully pixel-wise annotated RGB images as a supplement to the public dataset *Materials in Context* (MINC).

## Conclusions

The eight articles in this e-book show a wide range of state-of-the-art technologies and multidisciplinary approaches based on different use case scenarios. Each article shows the latest research progress and actively discusses the current technological problems. Through this article collection we can share the common issues and future perspectives. we expect this would contribute to make a breakthrough and promote optimal integration of systems from different fields of science and technologies.

